# Living in ancestrally diverse states of the United States is associated with greater vagal tone

**DOI:** 10.3389/fpsyg.2022.1068456

**Published:** 2023-01-12

**Authors:** Ethan G. Harrod, Ilan Shrira, Jared D. Martin, Paula M. Niedenthal

**Affiliations:** ^1^Department of Psychology, The University of Wisconsin-Madison, Madison, WI, United States; ^2^Department of Psychology, Pennsylvania State University, State College, PA, United States; ^3^OptumLabs, Minnetonka, MN, United States

**Keywords:** historical heterogeneity, heart rate variability, culture, ancestral diversity, vagal tone

## Abstract

Historically, exposure to dissimilar others (“strangers”) was a physiologically arousing event—resulting in avoidance, distrust, and even conflict. Despite this, contemporary migration patterns are increasing intergroup contact. What gives rise to an individual’s ability to regulate their arousal such that social engagement with outgroup members is possible? We propose that cultural practices that evolve in ancestrally diverse, compared to ancestrally homogeneous, societies provide more opportunities for society members to engage in emotion regulation. This regulatory exercise, in turn, promotes higher vagal tone—a physiological indicator of one’s ability to effectively manage arousal in social interaction. In a secondary analysis of data from the MIDUS 2 Biomarker Project, we find that the ancestral diversity of the states of the United States significantly predicts the average vagal tone of the state’s citizens. The findings suggest that social context is associated with predictable and significant adaptations of human physiology over individual lifetimes.

## 1. Introduction

The emergence of a globalized economy as well as increases in international migration has altered the world’s demographic landscape such that contact with people from different cultural and linguistic backgrounds has become a common feature of social life. In some regions of the world, defined at multiple geo-political levels (e.g., countries and states of countries), this trend is historically recent. In other regions, the intermingling of people from different cultures and linguistic groups has occurred continuously over a long history. With respect to evolved human predispositions, the intermingling of different peoples—intergroup contact—is a social challenge because humans largely prefer homogeneity. That is, people are generally more attracted to and comfortable around people similar to themselves (Moreland and Beach, 1992; McPherson et al., 2001). Contact with dissimilar people (“strangers”) typically elicits higher sympathetic nervous system arousal (e.g., stress and anxiety) relative to contact with familiar people (Blascovich et al., 2001; Mendes et al., 2002). Considering the trend toward cultural intermingling, a question urgently in need of an answer is what gives rise to the ability to successfully tolerate and socially engage with diverse others?

Here, we develop and test the idea that living in ancestrally diverse societies is associated with adaptations of human physiology, specifically of the vagal system, which in theory supports the ability to regulate sympathetic arousal such that social engagement is tolerable. We propose that the cultural practices and values that represent solutions to the challenges of living in ancestrally diverse populations over generations constitute opportunities to regulate arousal through the activation of the parasympathetic nervous system. Repeated exercise of parasympathetic control—*via* behavioral and emotional regulation during social interactions—should give rise to greater average vagal tone, an indicator of individual differences in regulatory control exerted by the parasympathetic nervous system. In the reported study, we test these ideas by examining the relationship between the ancestral diversity of the states of the United States and the average vagal tone of the states’ citizens, controlling for other factors known to explain significant variance in vagal tone.

Beginning roughly 500 years ago, innovations in cartography, shipbuilding, and navigation supported unprecedented waves of migration around the globe (Diamond, 1997). The distribution of the migration was, however, uneven. Some regions (e.g., present-day Brazil and the United Kingdom) became host to migrants from many different countries and, as a result, members of their populations had frequent contact with cultural outgroup members over history. Conversely, other regions (e.g., present-day Japan and Norway) were recipients of far fewer migrants (Putterman and Weil, 2010). Members of their populations therefore had very little contact with cultural outgroup members, or their norms and practices. Differences in the migratory history of regions are captured by a socio-ecological variable, ancestral diversity (Putterman and Weil, 2010), which can be quantified by the number of individual source countries that contributed to at least 0.5% of the current population of a given country since 1500 AD (Wood et al., 2016).

Importantly, life in ancestrally diverse societies is very different from life in less ancestrally diverse societies (Niedenthal et al., 2017). First, the former societies present the unique challenge of regularly navigating interactions with people very different from the self. Throughout human history, strangers and dissimilar people (i.e., outgroup members); on the rare occasions they were encountered, were viewed with suspicion, and were attacked or avoided (Neuberg and Schaller, 2016). Recent meta-analyses have noted that the prospect of interacting with outgroup members still produces negative affect and anxiety (Toosi et al., 2012), and the encounters themselves can trigger automatic threat-like responses indicated by sympathetic nervous system arousal, amygdala activation, and excessive cardiac reactivity (Phelps et al., 2000; Blascovich et al., 2001; Mendes et al., 2002). Second, ancestrally diverse societies are more unpredictable, meaning that it is harder to anticipate a social partner’s behavior, feelings, or goals (Niedenthal et al., 2019). Indeed, indicators of the ancestral diversity of countries of the world are negatively correlated with the tightness of prevalent social norms and positively correlated with present-day relational mobility, suggesting that norms and social ties in ancestrally diverse societies tend to be less rigid (Niedenthal et al., 2019). As noted by Niedenthal and colleagues, social unpredictability can be reduced if people make their internal states explicit through eye contact and nonverbal communication. Consistent with this claim, recent studies demonstrate that ancestral diversity positively predicts display rules for the overt expression (versus suppression) of emotion, cross-cultural recognizability of facial expressions, and the frequency of use of smiles of reward and affiliation (Rychlowska et al., 2015; Niedenthal et al., 2018, 2019). Importantly, the exchange of non-verbal signals such as eye contact and facial expressions, are themselves social challenges in that they are arousing and require emotion regulation.

It must also be noted that the development of such norms and practices requires long periods of time over which they can be transmitted generationally and evolve (Mesoudi, 2016). One potential consequence of this is that the alleviation of social tensions that often accompany initial exposure to diverse others (e.g., increases in outgroup prejudice and conflict; Esteban et al., 2012), may require long-term exposure to outgroup members in order for adaptive social strategies to evolve within a culture. Indeed, this idea is supported by recent work demonstrating that, while short term-exposure to diversity is associated with increases in outgroup prejudice and discrimination, long-term exposure to diversity is associated with decreases in prejudice and discrimination toward outgroup members (Ramos et al., 2019) In the context of ancestral diversity, this idea is supported by evidence that the effects of ancestral diversity on social cues and emotional expressivity are distinct from the impact of present-day diversity—quantified as the number of individual source countries which have contributed to at least 0.5% of a region’s population from 2000 to 2010 years (Niedenthal et al., 2018).

To summarize, societies defined by high vs. low ancestral diversity are very different social contexts for human development. The practice and norm characteristics of the former entail frequent experiences (e.g., eye contact, explicit displays of emotion, and numerous encounters with outgroup members) that require effective control over sympathetic arousal to successfully engage with individuals different from the self. Moreover, there is evidence to support the claim that frequent experiences of social challenges only result in socially beneficial practices and norms when afforded enough time for cultural evolution to occur.

Unlike species with phylogenetically older vagal systems, in humans, the vagal system governs two distinct strategies when responding to environmental challenges, particularly social ones (Porges, 1995, 2003, 2007). When threat levels are perceived to be high, the vagus’ influence is suspended or reduced, sympathetic arousal is uninhibited, and bodily resources needed to take appropriate action (e.g., fight or flight) in response to the threat are mobilized. When the environment is perceived as non-threatening, vagal activation can serve to rapidly “brake” the sympathetic nervous system’s influence on the heart to reduce activity of the hypothalamic–pituitary–adrenal axis and generate a calm state that enables successful social engagement (Porges, 2007). In contexts in which behavioral practices and norms involve activation of sympathetic arousal, the application of the vagal “brake” must occur frequently and accurately.

The efficacy and accuracy of the vagal system’s ability to “step on the brakes” at the level of the heart are referred to as vagal tone (Porges et al., 1996). Vagal tone varies across individuals and higher levels are associated with positive social outcomes including the tendency to engage in prosocial behavior and the ability to respond flexibly to environmental conditions (Kok and Fredrickson, 2010; Geisler et al., 2013). Children with greater vagal tone are less aggressive and less likely to exhibit behavioral control problems than children with lower vagal tone (Porges et al., 1996; Beauchaine et al., 2007). And adults with greater vagal tone have lower social anxiety (Porges et al., 1996), less defensiveness (Alvares et al., 2013), better emotion recognition (Movius and Allen, 2005), and stronger feelings of social connection when interacting with others (Colzato et al., 2017).

Critically for the present account, experiences of emotion regulation can reliably lead to temporary increases in an individual’s average, baseline vagal tone (Butler et al., 2006; Colzato et al., 2017), and, in theory, occur over medium timescales (e.g., ontogeny) if repeated. We therefore reasoned that if the norms and practices which evolved in societies with high ancestral diversity require frequent regulation of emotions and arousal, members of such societies should have, on average, greater vagal tone than members of societies low in ancestral diversity. For the present study, we accessed measures of high-frequency heart rate variability (HF-HRV) from the Midlife in the United States (MIDUS 2) Biomarker Project as an indicator of vagal tone. We then tested the potential relationship between the average HF-HRV of citizens of the U.S. and a measure of the ancestral diversity of the state in which they reside. We expected to observe a significant positive relationship, even when controlling for individual and state-level health and economic status.

Our account also holds that the adaptive norms and practices designed to successfully navigate the social challenges of diverse living may take multiple generations to fully develop, and benefit a population. Put another way, in contexts in which cultural practices have had insufficient time to evolve, initial interactions with diverse others may be linked to poor arousal regulation—indexed *via* vagal tone. We assessed this claim by also testing the potential relationship between the average HF-HRV of citizens of the U.S. and the present-day diversity of the state in which they reside while controlling for that state’s ancestral diversity as well as the other correlates of vagal tone. We expected to find no relationship or a negative relationship between present-day diversity and vagal tone—highlighting an important distinction between the effects of historical exposure to diversity and short-term exposure to diversity on the adaptation of one’s physiology.

## 2. Materials and methods

### 2.1. Ancestral diversity and present-day diversity

**Figure 1:**
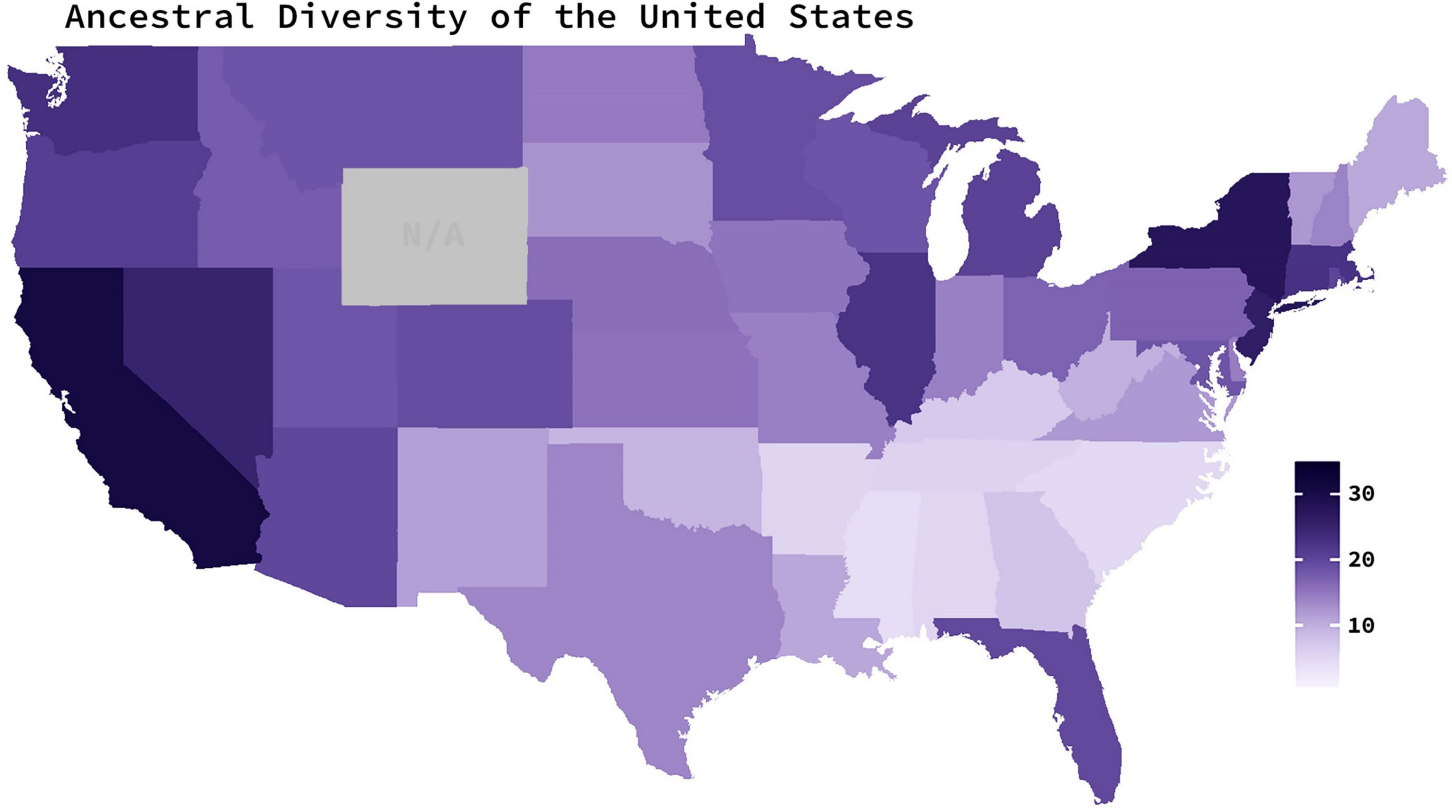
Ancestral diversity of the United States. Average number of foreign countries which have contributed to at least 0.5% of a state’s population since 1850. Darker colors indicate a larger number of source countries (operationalized as high ancestral diversity). Scores are based on decennial census data from 1850 to 2010. Gray indicates missing data.

Ancestral diversity scores for each U.S. state were operationalized as the average number of source countries that have contributed to at least 0.5% of states' populations from 1850 to 2010. Scores from 1850 to 2000 were collected from decennial census-provided estimates available for each state (U.S. Bureau of the Census, 1999; United States Census Bureau, 2003). 1850 was chosen as the starting point for these data as it marked the first U.S. census to collect data on each member of the household—including each resident’s country of birth (De Bow, 1853). Scores from 2010 were retrieved from the 2010 American Community Survey—a survey conducted by the U.S Census Bureau (United States Census Bureau, 2018)—as a replacement to the decennial censuses. Estimates of the number of source countries contributing to at least 0.5% of a state’s population were only available from 1860 onward for Kansas, North Dakota, Nebraska, Nevada, South Dakota, and Washington; from 1870 onward for Arizona, Colorado, Idaho, and Montana; and from 1890 onward for Oklahoma. Alaska and Hawaii were excluded from the calculations because census data for these states were only available from 1960 onward and thus would not indicate a long history of heterogeneity. Ancestral diversity scores from the 48 sampled states varied between 4.11 and 31.05 (*M* = 15.71, *SD* = 6.52; Figure 1.).

The present-day diversity score for each U.S. state was operationalized as the number of source countries which have contributed to at least 0.5% of the state’s population from 2000 to 2010. An estimate of this number was retrieved from the 2010 American Community Survey for 48 of the 50 states (all U.S. states excluding Hawaii and Alaska; United States Census Bureau, 2003). Present-day diversity scores varied between 10 and 40 (*M* = 24.93, *SD* = 8.22).

### 2.2. HF-HRV and demographics

We conducted analyses using data from three existing datasets. Baseline HF-HRV, demographic, and health characteristic data were originally collected during the Bioindicators project of the National Survey of Midlife Development in the United States (MIDUS 1) follow-up study (MIDUS 2). MIDUS 1 is a nationally representative sample of 7,108 individuals across 50,000 households selected *via* random telephone dialing (Brim et al., 2018). MIDUS 2 followed up with 5,895 of the original MIDUS 1 participants with a 30-min telephone survey (Ryff et al., 2019). Of these 5,895 participants, 1,255 were eligible, and subsequently selected, to participate in the Bioindicators project (Ryff et al., 2017). Participants (*n* = 1,255) who comprise the Bioindicators project had a mean age of 54.5 (*SD* = 11.7), were mostly White (78.3%), and 56.8% were female. Participants attended a 2-day hospital clinic visit during which they underwent cardiovascular, immune, neuroendocrine, and basic physical assessments. Continuous ECG and respiration recordings were taken during the entire 2-day visit and used to calculate a measurement of both low and HF-HRV for each participant. Complete details about how ECG and respiration data were collected, cleaned, used to calculate HF-HRV, and transformed can be found in the “Bioindicators in the MIDUS National Study Protocol” (Dienberg Love et al., 2010).

The present analyses utilized the HF-HRV data collected during a 6-min resting baseline period. Each participant’s baseline HF-HRV data were residualized on their sex, age, and BMI. These health-adjusted HF-HRV scores were then used to calculate cluster-means at the level of the state. The lack of individual-level data from Alaska, Hawaii, and Wyoming, as well as the inclusion of data from Washington DC (counted as a state here for the sake of uniformity in terms), resulted in 48 total “state-level” HF-HRV scores. All analyses that included HF-HRV as a variable were performed using these demographic-adjusted, state-level HF-HRV scores. Scores varied between −0.764 and 1.12 (*M* = −0.035, *SD* = 0.647), with larger scores representing a greater baseline HF-HRV. The number of observations per state varied between 1 and 213 (*M* = 22.79, *SD* = 32.96). Because of the large disparity in the number of observations per state, weighted least square regression, with weights determined by the number of observations, was used in all relevant analyses.

### 2.3. State health

State health scores were retrieved from the 2019 Opportunity Index (Opportunity Index, 2019). The Opportunity Index is an annual report which collects demographic, social, and economic data from individuals across all 50 US states. These data are used to determine a single Opportunity Index score for each state. The Opportunity Index calculates state health scores from three health indicators: The percentage of infants born weighing less than 5.5 pounds, the percentage of a state’s population (under the age of 65) without health insurance, and the number of deaths per 100,000 attributed to alcohol or drug poisoning, or suicide. Scores were then standardized, and reverse coded so that higher scores indicated greater health opportunities. State Health scores varied between 36.7 and 71.2 (*M* = 54.6, *SD* = 8.7).

## 3. Results

### 3.1. Data cleaning and outlier removal

All analyses reported in the present manuscript, unless otherwise stated; use state-level data from 46 of the 50 US states. HF-HRV and ancestral diversity data from Alaska, Hawaii, and Wyoming were not available and, thus, not included in the analyses. An evaluation of model assumptions as well as outlier analyses was conducted to check for observations with strong influence on our final models. Following field standards (Belsley, 1980), a DFBETA cutoff of 2/sqrt(*n*) (0.29) was set. All observations which exceeded this cutoff were classified of model outliers and not included in our final analyses. Both Rhode Island and Washington DC exceeded this cutoff (DFBETA >0.29) and were excluded from analyses. Our main findings are derived from analyses utilizing the outlier-adjusted dataset (i.e., excluding Rhode Island and Washington). However, we have also provided the output from our analyses when using the full dataset (i.e., a dataset including the aforementioned outlier states).

### 3.2. Ancestral diversity and HF-HRV

**Figure 2:**
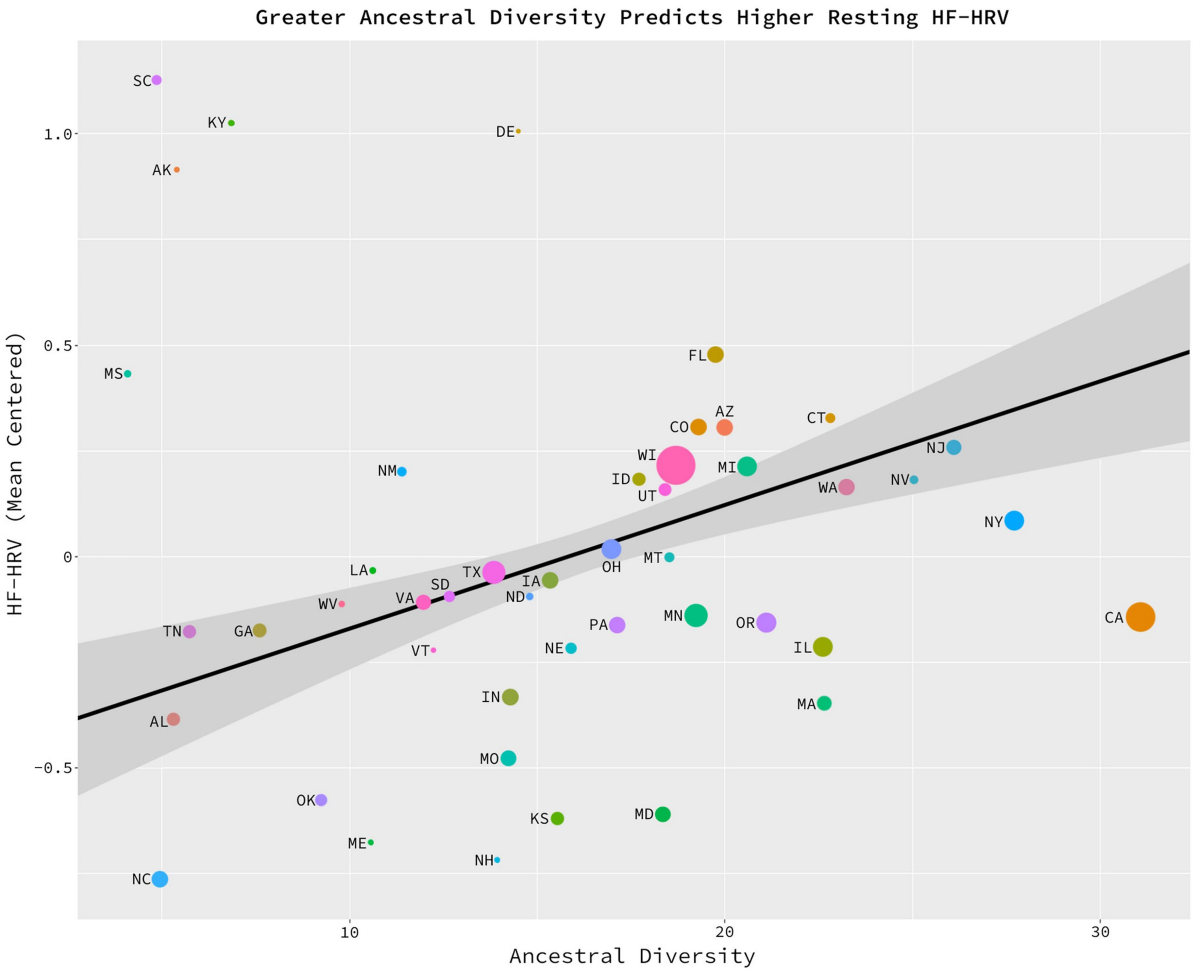
Greater ancestral diversity related to higher HF-HRV. Significant positive relationship between ancestral diversity and the average, baseline HF-HRV of the states’ citizens, *b* = 0.029, *sd_b* = 0.435, *t*(42) = 2.291, *p* = 0.027, η_p_^2^ = 0.11, CILO = 0.003, CIHI = 0.055. On the *x* axis, the average number of foreign countries which have contributed to at least 0.5% of a state’s population since 1850. On the *y* axis, a mean-centered average of baseline, HF-HRV residualized on health, age, and sex. This figure is generated from a data set consisting of 46 data points. Each data point represents one of the U.S. states (excluding Alaska, Hawaii, Rhode Island, and Wyoming). The size of each data point represents the number of observations per state from which the HF-HRV scores were calculated.

The potential relationship between ancestral diversity and HF-HRV was assessed with a weighted least squares, multiple regression model in which health-adjusted HF-HRV (i.e., HF-HRV while statistically controlling for the effects of individual health, sex, age, and BMI) was regressed on ancestral diversity while statistically controlling for present-day diversity and state health. Model weights were based upon the number of observations per state (*M* = 23.74, *SD* = 33.36). As predicted, there was a significant linear relationship between ancestral diversity and HF-HRV, *b* = 0.029, *sd_b* = 0.435, *t* (42) = 2.291, *p* = 0.027, η_p_^2^ = 0.11, CILO = 0.003, CIHI = 0.055 (Figure 2). Residents of states high in ancestral diversity (e.g., Illinois, New Jersey, and Michigan) had greater baseline HF-HRV than did residents of ancestrally homogeneous states (e.g., Alabama, Georgia, and Tennessee). The full model accounted for 13% of the total variance (*R^2^* = 0.134) in HF-HRV scores.

### 3.3. Present-day diversity and HF-HRV

**Figure 3:**
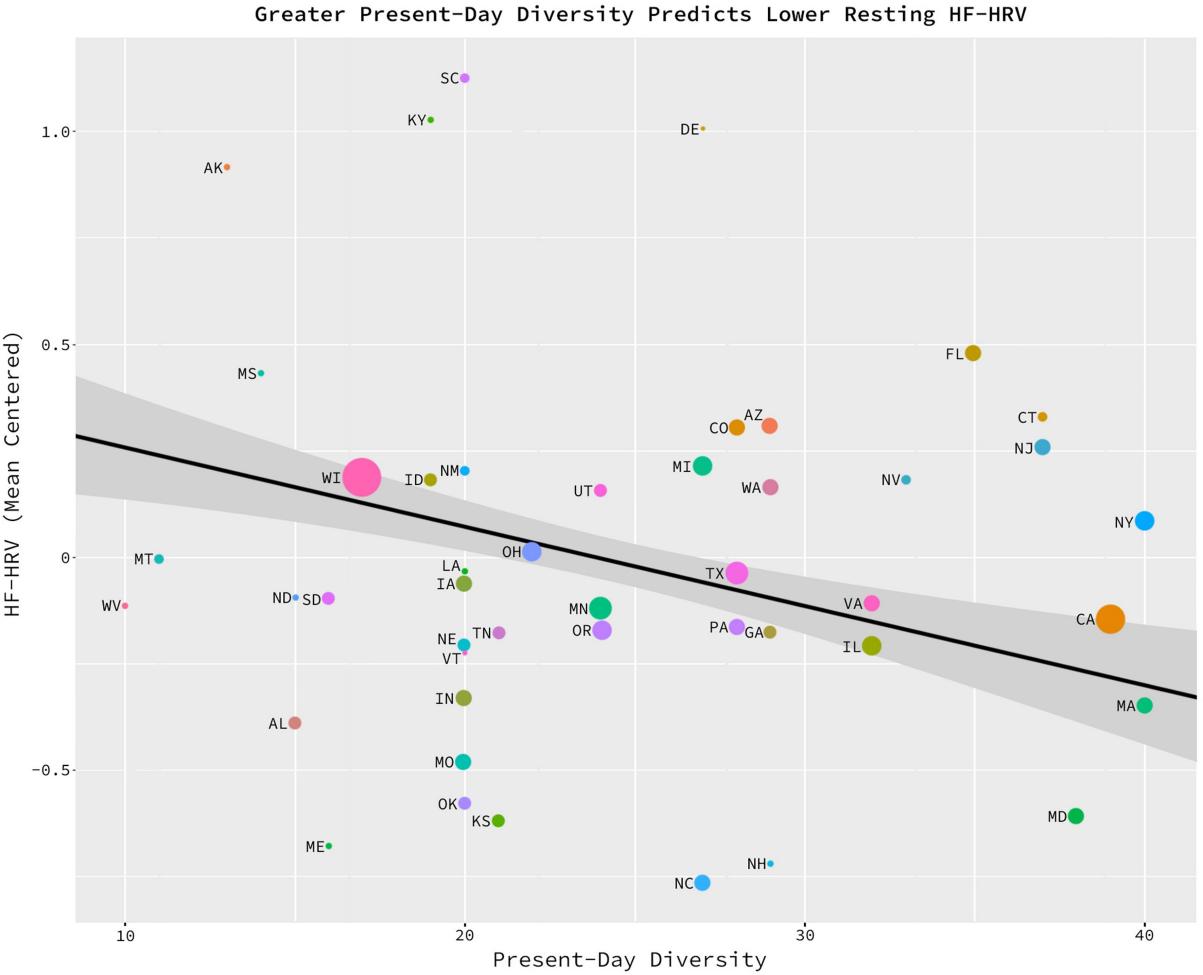
Greater present-day diversity related to lower HF-HRV. Significant negative relationship between present-day diversity and the average, baseline HF-HRV of the states’ citizens, *b* = −0.018, *sd_b* = −0.277, *t* (42) = −2.299, *p* = 0.0265, η_p_^2^ = 0.11, CILO = −0.034, CIHI = −0.002. On the *x* axis, the average number of foreign countries which have contributed to at least 0.5% of a state’s population since 2010. On the *y* axis, a mean-centered average of baseline, HF-HRV residualized on health, age, and sex. This figure is generated from a data set consisting of 46 data points. Each data point represents one of the U.S. states (excluding Alaska, Hawaii, Rhode Island, and Wyoming). The size of each data point represents the number of observations per state from which the HF-HRV scores were calculated.

The potential relationship between present-day diversity and HF-HRV was also assessed through the analysis of the previously introduced statistical model. As predicted, analysis revealed a significant, negative relationship between present-day diversity and HF-HRV, *b* = −0.018, *sd_b* = −0.277, *t* (42) = −2.299, *p* = 0.0265, η_p_^2^ = 0.11, CILO = −0.034, CIHI = −0.002 (Figure 3). On average, individuals living in states in which the present-day population has been informed by a greater number of foreign countries since 2010 (i.e., more heterogenous and present-day state populations) have lower baseline HF-HRV as compared to individuals residing in states in which the present-day population has been informed by fewer countries since 2000–2010 (i.e., more homogeneous and present-day state populations). This relationship held above and beyond the effects of ancestral diversity, state health (i.e., health insurance coverage, low birth weight, and substance-related deaths), and individual health (i.e., BMI, age, and Sex).

### 3.4. Control variables and outlier impact

In light of the present study’s limitations in power (see our limitations section for more information), the selection of control variables was done conservatively and in observation of the potential impact that the careless inclusion of control variables can have on the conclusions drawn from our analyses (Spector and Brannick, 2011). As such, our choice of control variables was primarily informed by existing guidelines for the analysis of HF-HRV data (Quintana and Heathers, 2014). This included both state health and individual health (accounted for by residualizing HF-HRV scores on individual health variables such as BMI, sex, and age). Despite its inclusion as a control variable, the effect of state health on HF-HRV, while statistically accounting for all other predictors in the model, was not significant (*p* = 0.064). Additionally, preliminary analyses revealed that state-level demographic variables such as state education and economic health were not significantly related to HF-HRV (*p* > 0.10). This fact, in combination with the previously mentioned conservative approach to the implementation of statistical controls, resulted in the decision to not include these variables in our final model.

Analysis of the abovementioned statistical model while using the full dataset (i.e., data including the previously identified outlier states) yielded similar results. There was a significant, positive, linear relationship between HF-HRV and ancestral diversity, *b* = 0.029, *sd_b* = 0.291, *t* (44) = 2.291, *p* = 0.032, η_p_^2^ = 0.10, CILO = 0.002 CIHI = 0.055, as well as a significant, negative, linear relationship between HF-HRV and present-day diversity, *b* = −0.018, *sd_b* = −0.185, *t*(44) = −2.226, *p* = 0.0312, η_p_^2^ = 0.10, CILO = −0.035 CIHI = −0.001.

## 4. Discussion

Despite historically fearing and avoiding individuals considered to be outgroup members (e.g., of different ethnic, racial, or religious groups), humans now find themselves in societies that grow more diverse by the day. Previous work has shown that the intermingling of heterogeneous populations, over long history, gives rise to practices and values that support successful communication and cooperation (Niedenthal et al., 2019). Results of the present study suggest further that these practices and values constitute a social context associated with specific adaptations of human physiology. We find that citizens who reside in states of the U.S. that are high in ancestral diversity have greater average vagal tone, an indicator of effective emotion regulation, than citizens who reside in states lower in ancestral diversity. This effect persists when controlling for factors such as individual and state health—both of which can have a substantial relationship to vagal tone (Karason et al., 1999; Aziz et al., 2012; Hill et al., 2015; Koenig and Thayer, 2016).

The negative relationship between present-day diversity and vagal tone—while controlling for ancestral diversity—suggests an important distinction between the effects of long-term (ancestral) and short-term (present-day) exposure to diversity. We posit that the cultural norms and practices which evolved in ancestrally diverse populations—greater emotional expressivity, behavioral regulation, and emotional regulation—are specific to social interactions with diverse others for whom those norms were necessitated. For example, norms and practices initially designed to facilitate healthy interactions with large number of immigrants from Germany and Norway in Wisconsin *circa* 1850 may not extend to, or be effective during, social interactions with immigrants from India or Mexico in present-day Wisconsin. Indeed, past research has shown that large-scale changes in cultural norms require transmission across multiple generations (Mesoudi, 2016). Moreover, when interacting with diverse others for whom adaptive cultural norms have had insufficient time to evolve, it is possible that the norms and cultural practices for interacting with these newer out-group members lead to increased conflict—an outcome that has been linked to initial increases in diversity (Esteban et al., 2012). Future work will test the theory that the negative link between present-day diversity—despite the positive relationship with ancestral diversity—may be explained by differences between the countries which historically immigrated to and contributed to a state’s population and the countries which are presently immigrating and contributing to a state’s population.

### 4.1. Limitations

Although the present theory is novel and the findings are robust, claims about the cause of the observed relationship must be made with caution. It is possible that individuals with greater vagal tone were more likely to migrate to states that became ancestrally diverse. It is also possible that a third variable is responsible for our findings. For example, immigrants from many different countries with diets high in fish oil—the consumption of fish is linked to greater vagal tone (Mozaffarian et al., 2008)—may have migrated to the Great Lake States or other coastal regions of the U.S. due to a desire to replicate their diet in the New World. Thus, these populations would have been both diverse and high in average vagal tone without one variable causing the other. The issue is a complex one, to which future experimental, as compared to cross-sectional, studies may offer greater insight.

Additionally, the majoritively White populations (78.3%) from the MIDUS 2 Bioindicators project limits the generalizability of the present findings. The social contexts in which Americans lived from 1850 to 2010, and the practices required to successfully navigate those contexts have been—and still are—irrefutably different for people of color, as compared to Whites. Consequentially, it cannot be assumed that the theoretical motivation behind the present analyses is equally compelling for populations which have dealt and are dealing with the added social—and physiological (Williams and Mohammed, 2013)—threat of discrimination and systemic racism. Nor can it be assumed that the present findings hold true populations of color. Indeed, a 2015 meta-analysis revealed that, on average, Black Americans, as compared to White Americans, possess greater baseline HRV (Hill et al., 2015). It has since been posited that this finding—dubbed the “African American Vagal Advantage”—may be linked to the experience and anticipation of racial discrimination (Snijders, 2005). Future work aims to address this limitation by examining the relationship between ancestral diversity and HRV in majoritively Black populations and by examining the potential moderating effect of participant race and state-level indicators of historic and present-day discrimination.

It should also be noted that the number of observations (*n* = 1,255) is not evenly distributed across the 48 sampled states. This uneven distribution is addressed and accounted for in our statistical models through the use of a weighted analysis. Similarly, it must be noted that statistical power for a level-2 analysis—such as the analyses conducted in the present study—is derived from the number of level-2 observations (Snijders, 2005). Because the maximum possible sample size is 51 (i.e., the number of U.S. states and Washington D.C.), the study’s potential power is hard capped at 0.66. With a sample size of 46 (following the removal of the two outlier states, Rhode Island and Washington), the present study has a statistical power of 0.63. Certainly, this limit in power hinders the extent to which firm conclusions can be confidently drawn from the present findings. As such, these findings should be considered skeptically. Future studies are aimed at replicating the present study’s findings on an international scale. Sampling from countries, rather than states, would remove the cap of 51 possible observations and, in turn, the limitations in power.

Finally, the present findings only provide information about state-level effects and may not generalize to the individual-level. As such, more precise claims about variation at the individual-level or variation within the states cannot be made from these findings but instead stand as valuable questions to be explored by future research and may also lend insight into questions of directionality.

### 4.2. Conclusion

The present work serves as a foundational step for multiple lines of research. The findings imply that short-term exposure to greater diversity is not sufficient in promoting adaptive physiological changes and, in turn, greater social tolerance. Rather, such physiological changes may require continued, long-history exposure to the practices and values codified in the cultures that arise over time in ancestrally diverse contexts. This nuance raises an interesting question of how to foster lasting social tolerance in the face of diversity despite its negative short-term impact on vagal tone. Further research will not only further our knowledge of how cultural differences arise but will also serve as a useful guide as outgroup interactions inevitably become more commonplace. Within cultural psychology and the study of cultural differences, the present work provides a newfound physiological mechanism for the well-documented cultural differences in emotional expression, emotional regulation, stress regulation, and outgroup prejudice. Finally, within the field of physiology, the noted long-history changes and regional differences in vagal tone will pave the way for research dedicated to understanding well-documented, but seemingly paradoxical, group-level phenomena such as the African American vagal advantage or the Female vagal advantage (Hill et al., 2017; Shaffer and Ginsberg, 2017).

The value of the present work lies in its role as the grounds for a broader theoretical relationship between cultural differences in social behavior and human physiology. It not only demonstrates that where we live and what cultures we are a part of shape our physical and social processes, but ultimately sets the stage for research dedicated to understanding how to cultivate and maintain greater social tolerance—knowledge which is crucial in our ever-shrinking world.

## Data availability statement

The original contributions presented in the study are included in the article/supplementary material, further inquiries can be directed to the corresponding author.

## Ethics statement

The studies involving human participants were reviewed and approved by the University of Wisconsin-Madison IRB. Written informed consent for participation was not required for this study in accordance with the national legislation and institutional requirements.

## Author contributions

EH: first authorship, formal analysis, and visualization. EH, IS, JM, and PN: conceptualization. EH and JM: methodology. JM and IS: data curation. PN: supervision. IS: writing—original draft. EH and PN: writing—review and editing. All authors contributed to the article and approved the submitted version.

## Funding

This work was supported by Cattell Sabbatical Award MSN206694 (PN).

## Conflict of interest

JM was employed by OptumLabs.

The remaining authors declare that the research was conducted in the absence of any commercial or financial relationships that could be construed as a potential conflict of interest.

The reviewer MC declared a shared affiliation with the author IS to the handling editor at the time of review.

## Publisher’s note

All claims expressed in this article are solely those of the authors and do not necessarily represent those of their affiliated organizations, or those of the publisher, the editors and the reviewers. Any product that may be evaluated in this article, or claim that may be made by its manufacturer, is not guaranteed or endorsed by the publisher.
